# The Microscope: Its History, Construction, and Application

**Published:** 1857-07

**Authors:** 


ART. VIII.—*The Microscope: its History, Construction, and Application.* By JABEZ HOGG, M.R.C.S. London.

OUR Transatlantic neighbors have so often indulged in whining complaints of the appropriation of their literary labors by others, that it has become a sort of stereotyped criticism upon American publications, no matter how faithfully they may have given credit to their contemporaries when occasion required a reference to their productions. In the instance, however, now before us, the boot is on the other foot. The book of Mr. Hogg has, no doubt, considerable merit as a compilation; and in giving it this title, we mean no disrespect, for it is our opinion, that no useful book on the microscope has been, or can be written, which is not, to a great extent, a compilation. Even Quekett, whose work is regarded as a standard, is largely indebted to his predecessors, especially the works of Pritchard and Goring. Yet that some notice should be taken of their researches, is certainly due to those who have gone before, no matter to what nation they belong. In the work of Mr. Hogg, this common principle of courtesy, and we might add, of honesty, is entirely ignored in reference to an American author, the first, we believe, in this field of research in this country, Dr. Joseph H. Wythes, from whose book, "The Microscopist," whole paragraphs, and nearly an entire chapter, have been copied *verbatim et literatim*, without the slightest acknowledgment or reference—a *Hogg-ish* proceeding certainly.

That we have not made this charge without foundation, will be evident from a comparison of the following paragraphs from the works in question:

*Hogg on the Microscope.*

"Mr. Goadby has succeeded in supplying to microscopists a ready, cheap, and effectual means for mounting animal structures with the greatest possible ease and security. The following are his formulæ:

"Take for No. 1 solution, bay salt, 4 oz.; alum, 2 oz.; corrosive sublimate, 2 grs.; boiling water, 1 quart; mix.

"For No. 2 solution, bay salt, 4 oz.; alum, 2 oz.; corrosive sublimate, 4 grs.; boiling water, 2 quarts; mix.

"The No. 1 is too strong for most purposes, and should only be employed where great astringency is needed to give form and support to very delicate structures. No. 2 is best adapted for permanent preparations; but neither should be used in the preservation of animals containing carbonate of lime (all the mollusca), as the alum becomes

*Wythes' Microscopist.*

"Dr. Goadby has devoted much attention to this subject, and has succeeded in supplying to the microscopist a ready, cheap, and effectual means for mounting animal structures with the greatest possible ease and security. Dr. G. received a gold medal from the Society of Arts for his invention. He has kindly furnished me with the following description of his different preserving fluids:

"'A 1. Bay salt (coarse sea salt), 4 oz.
Alum, 2 oz.
Corrosive sublimate, 2 grs.
Boiling water, 1 quart.

"'A 2. Bay salt, 4 oz.
Alum, 2 oz.
Corrosive sublimate, 4 grs.
Boiling water, 2 quarts.

"'The A 1 fluid is too strong for decomposed, sulphate of lime is precipitated, and the preparation spoiled. "For such use the following," &c. (p. 83.)

most purposes, and is only to be employed where great astringency is required to give form and support to delicate structures.

"'The A 2 fluid may be very extensively used, and is best adapted for permanent preparations; but neither of these fluids should be used in the preservation of animals possessing any carbonate of lime (all the mollusca), as the alum becomes decomposed, and the sulphate of lime is formed and precipitated, and the animal spoiled. For such use the B fluid,'" &c. (p. 57.)

Mr. Hogg, it seems to us, has taken more trouble in endeavoring to improve the style of the above than would have sufficed to have given credit for the extract. Again, we pursue the comparison:

*Hogg on the Microscope*, p. 84.

"For minute injections, the most essential instrument is a proper syringe. This is usually made of brass, of such size that the top of the thumb may press on the button at the top of the piston-rod when drawn out, while the body is supported between the two fingers. Fig. 65 represents the syringe."

*Wythes' Microscopist*, p. 159.

"For minute injection (as it is called), the most essential instrument is a proper syringe. This should be made of brass, of such a size that the top of the thumb may press on the head or handle of the piston-rod when drawn out, while the body is supported by two of the fingers of the same hand. Fig. 50 represents a syringe, with which I have succeeded in making some excellent preparations."

The description of the syringe and the woodcuts are identical in the two works.

"In addition to the syringe, a large tin vessel, to contain hot water, with two or three lesser ones fixed in it, for the injections, will be found useful."

"In addition to the syringe, a large tin vessel, to contain hot water, with two or three lesser ones fixed in it for the injections, will be found useful."

Mr. Hogg gives a woodcut of such a vessel, and its description, with another cut and description of an aneurism needle, is the only part of his chapter on minute injections which is not extracted verbatim from Dr. Wythes' work, without the slightest acknowledgment. Not contented with this dishonorable conduct, Mr. Hogg mutilates his extracts so as to rob our townsman, Dr. Goddard, of his due credit, and also, so as to make certain remarks of Dr. Goadby appear like recommendations of his own. The following is the proof:

*Hogg*, page 87.—" Another process, which may be termed the chemical process, was published in the *Comptes Rendus,* 1841, as the invention of M. Doyere. According to this an aqueous solution," &c.

"I would recommend that the slips of glass employed," &c. page 88. " Although highly desirable, as the demonstrator of the capillaries of *normal* tissues, I do not think this kind of injection fitted for morbid preparations," &c.

*Wythes*, page 163.—" Another process, which may be termed the chemical process, was published in the *Comptes Rendus,* 1841, as the invention of M. Doyere, though the credit of first suggesting it is due to Dr. Goddard, of Philadelphia. According to this, an aqueous solution," &c.

In the " Microscopist," these paragraphs, with others of a similar kind, are quoted from a paragraph furnished by Dr. Goadby.

Again:

*Hogg*, p. 88.—" Still another plan has been suggested. It consists in adding a quantity of sulphuric ether to the finely levigated coloring matter, which is also first ground or mixed with linseed oil, in the manner employed by painters. Upon this plan (as well as upon the last named), some beautiful injections of the smallest capillaries are made; but sometimes it is a failure, owing to the too rapid evaporation of the ether, and the clogging up of the vessels from the early deposition of the solid coloring matter. Perhaps a solution of gum mastic in ether, colored with fine vermilion, will answer the purpose better."

*Wythes*, p. 166.—" Still another, and in some respects a more certain and convenient plan has been employed by Dr. Goddard, of Philadelphia. It consists in adding a quantity of sulphuric ether to the finely levigated coloring matter, which is also first ground or mixed with linseed oil, in the manner employed by painters. Upon this plan (as well as upon the last named), I have succeeded in making some beautiful injections of the smallest capillaries, yet I have sometimes failed, owing to the too rapid evaporation of the ether, and the clogging up of the vessels from the early deposition of the coloring matter. I have also observed, that after the ether has evaporated from the vessels, the particles of coloring material cohered with too little tenacity, so that on putting a section of injected tissue into turpentine, &c., the color has been washed out from the cut ends of the larger vessels. Perhaps a solution of gum mastic, &c., in ether, colored with fine vermilion, &c., will answer the purpose better."

We can scarcely trust our pen to express our utter contempt for the conduct of which Mr. Hogg has been guilty, and dismiss the subject with the above *exposé* of his plagiarism.

